# Relationship between total cholesterol level and tuberculosis risk in a nationwide longitudinal cohort

**DOI:** 10.1038/s41598-021-95704-1

**Published:** 2021-08-10

**Authors:** Yong Suk Jo, Kyungdo Han, Dahye Kim, Jung Eun Yoo, Yuji Kim, Bumhee Yang, Hayoung Choi, Jang Won Sohn, Dong Wook Shin, Hyun Lee

**Affiliations:** 1 Division of Pulmonary, Allergy, and Critical Care Medicine, Department of Internal Medicine, Hallym University Kangdong Sacred Heart Hospital, Seoul, Korea; 2 Department of Statistics and Actuarial Science, Soongsil University, Seoul, Korea; 3 Department of Biostatistics, The Catholic University of Korea, Seoul, Republic of Korea; 4 Department of Family Medicine, Healthcare System Gangnam Center, Seoul National University Hospital, Seoul, Republic of Korea; 5 Division of Endocrinology and Metabolism, Department of Internal Medicine, Jeonbuk National University Hospital, Jeonju, Korea; 6 Division of Pulmonary and Critical Care Medicine, Department of Internal Medicine, Chungbuk National University Hospital, Chungbuk National University College of Medicine, Cheongju, Korea; 7 Division of Pulmonary, Allergy, and Critical Care Medicine, Department of Internal Medicine, Hallym University Kangnam Sacred Heart Hospital, Hallym University College of Medicine, Seoul, Korea; 8 Division of Pulmonary Medicine and Allergy, Department of Internal Medicine, Hanyang University College of Medicine, 222-1, Wangsimni-ro, Seongdong-gu, Seoul, 04763 Korea; 9 Department of Family Medicine/Supportive Care Center, Samsung Medical Center, Sungkyunkwan University School of Medicine, 81 Irwon-Ro, Gangnam-gu, Seoul, 06351 Korea; 10 Department of Clinical Research Design and Evaluation, SAIHST, Sungkyunkwan University, Seoul, Korea

**Keywords:** Diseases, Medical research, Risk factors

## Abstract

The association between the total cholesterol level and tuberculosis (TB) risk has been controversial. Our study aimed to evaluate whether total cholesterol level can predict the risk of TB. Data from 5,000,566 subjects who participated in a health screening exam in 2009 were investigated using the Korean National Health Insurance Service database (2009–2018). Cox hazard regression analyses were used to evaluate TB risk according to the quartile of total cholesterol levels. During an average of 8.2 years of follow-up, 32,078 cases of TB occurred. There was a significant inverse association between the total cholesterol level and TB risk. Compared with subjects in the highest quartile, those in the lowest quartile had a 1.35-fold increased TB risk (95% confidence interval = 1.31–1.39). The association between total cholesterol level and TB risk was more apparent in young subjects (age < 65 years), those without diabetes mellitus (DM), and those without obesity (*p* for interaction < 0.001 for age group, DM, and body mass index). Although there was a significant inverse association between total cholesterol level and TB risk in subjects who did not use a statin, no significant association was observed between the total cholesterol level and TB risk in subjects who used a statin. A low total cholesterol level was significantly associated with an increased risk of TB, even after adjusting for confounders, especially in patients younger than 65 years, those without DM or obesity, and those who did not use a statin.

## Introduction

Tuberculosis (TB) is one of the oldest diseases and remains a leading cause of death from an infectious disease^1^. In South Korea, the incidence and mortality rates for TB were 66 and 4.7 per 100,000 people, respectively, in 2018, which is five to six-fold higher than the average in the Organization for Economic Cooperation and Development (OECD) countries^2^. Although the occurrence of TB has been decreasing thanks to a nationwide, multifaceted effort, South Korea had the highest incidence and mortality rates for TB among OECD countries. Despite the historical prevalence of TB in Korea, socioeconomic development has made Korea a high-income country, which has substantially reduced the traditional risk factors for TB—poverty, undernutrition, and low body mass index (BMI)^3–5^. On the other hand, metabolic conditions (e.g., obesity, dyslipidemia, and diabetes mellitus [DM])^5–11^ which could alter the TB risk have increased in South Korea. Many developing countries with an intermediate–high TB burden face situations similar to that of South Korea; therefore, studies evaluating the relationship between metabolic diseases and TB risk will be important to those countries.

Cholesterol is a key component of the cell membrane and plays a crucial role in several metabolic pathways^12^. It has been suggested that altered lipid metabolism can affect susceptibility to TB. However, conflicting data about the relationship between the total cholesterol level and TB risk have been published^6–8^. Some researchers suggested that a low cholesterol level is associated with an increased risk of TB by showing that cholesterol levels decreased in patients with TB^6^. Supporting that view, studies showed that low cholesterol levels correlate with disease severity^13,14^. In contrast, other researchers suggested that low cholesterol levels in patients with TB are merely a consequence of the disease because TB treatment significantly increases low cholesterol levels^7,8^. Therefore, whether a low total cholesterol level is a contributing factor in TB or a consequence of the disease has been unclear. The relationship between a high cholesterol level and TB risk is also suggested. A positive association between a cholesterol-rich diet and TB risk in a cohort study of Singapore^15^ and study results indicating that latent TB treatment decreases the total cholesterol level^16^ suggest that a high cholesterol level might reflect a high TB risk status. In line with those results, hypercholesterolemia was shown to impair immunity to TB in animal models^9^.

Furthermore, cholesterol levels and TB risk are both affected by factors such as BMI^5^, DM status^10^, and statin use^17^, which makes understanding the association between them difficult. Therefore, studies evaluating the association between cholesterol and TB risk need to consider those factors. In this study, we aimed to identify the association between cholesterol level and the risk of TB using a nationwide cohort from South Korea while also considering the effects of BMI, DM, and the use of a statin on the association.

## Results

### Baseline characteristics

Table 1 summarizes the comparison of clinical features between the subjects who developed TB and those who did not. Subjects who developed TB were older (mean 56.1 years vs. 47.1 years, *p* < 0.001) and more likely to be male (58.9% vs. 54.6%, *p* < 0_._001) than those who did not. Compared with subjects who did not develop TB, those who developed TB had a lower BMI (mean 22.6 kg/m^2^ vs. 23.7 kg/m^2^, *p* < 0.001) and lower incomes (22.0% vs. 78.5%, *p* = 0.026). Subjects who developed TB had more comorbid conditions, including DM, hypertension, dyslipidemia, heart disease, stroke, chronic kidney disease (CKD), and chronic airway diseases (asthma or chronic obstructive pulmonary disease [COPD]) (*p* < 0_._001 for all comorbidities) than those who did not develop TB. In contrast, subjects who developed TB were less likely to do regular physical activity (17.3% vs. 18.2%, *p* < 0.001) than those who did not. The mean or median levels of total cholesterol (191.0 ± 37.9 vs. 195.1 ± 36.8 mg/dL), low-density lipoprotein (LDL) cholesterol (109.9 ± 34.8 vs. 113.2 ± 33.9 mg/dL), and triglycerides (110.7 [110.1–111.4] vs. 112.2 [112.2–112.3] mg/dL) were lower in subjects who developed TB than in those who did not (*p* < 0.001 for all variables). However, statin use was more common in subjects who developed TB than in those who did not (9.8% vs. 7.6%, *p* < 0.001).

**Table 1:** Demographic data of subjects.

	Total (N = 5,000,566)	Patients who did not develop TB (n = 4,968,488)	Patients who developed TB (n = 32,078)	*p* value
Age, years	47.2 ± 14.1	47.1 ± 14.1	56.1 ± 15.8	< 0.001
Sex, male	2,732,571 (54.7)	2,713,664 (54.6)	18,907 (58.9)	< 0.001
Body mass index, kg/m^2^	23.7 ± 3.2	23.7 ± 3.2	22.6 ± 3.1	< 0.001
Smoking status				
Never	2,983,062 (59.7)	2,964,482 (59.7)	18,580 (57.9)	< 0.001
Ex	712,535 (14.2)	707,910 (14.3)	4625 (14.4)	
Current	1,304,969 (26.1)	129,6097 (26.1)	8872 (27.7)	
Low income	1,075,880 (21.5)	78.5 (21.5)	78.0 (22.0)	0.026
Alcohol consumption				
No	2,585,695 (51.7)	2,567,446 (51.7)	18,249 (56.9)	< 0.001
Mild	2,020,311 (40.4)	2,009,501 (40.4)	10,810 (33.7)	
Heavy	394,560 (7.9)	391,542 (7.9)	3018 (9.4)	
Comorbid conditions				
Diabetes mellitus	436,055 (8.7)	430,767 (8.7)	5049 (15.7)	< 0.001
Hypertension	1,350,494 (27.0)	1,338,510 (26.9)	11,904 (37.1)	< 0.001
Dyslipidemia	909,576 (18.2)	903,271 (18.2)	6152 (19.2)	< 0.001
Heart disease	99,770 (3.2)	156,010 (3.1)	1703 (5.3)	< 0.001
Stroke	51,644 (1.6)	80,986 (1.6)	741 (2.3)	< 0.001
Chronic kidney disease, GFR < 30 mL/min/1_._73 m^2^	72,882 (1.5)	72,363(1.5)	519 (1.6)	< 0.001
Asthma or COPD	416,492 (8.3)	411,887 (8.3)	4689 (14.6)	< 0.001
Regular physical activity	907,094 (18.1)	901,780 (18.2)	5533 (17.3)	< 0.001
Lipid profile				
Total cholesterol level, mg/dL	195.1 ± 36.8	195.1 ± 36.8	191.0 ± 37.9	< 0.001
LDL cholesterol, mg/dL	113.2 ± 33.9	113.2 ± 33.9	109.9 ± 34.8	< 0.001
Triglycerides, mg/dL	112.2 (112.2–112.3)	112.2 (112.2–112.3)	110.7 (110.1–111.4)	< 0.001
HDL cholesterol, mg/dL	55.2 ± 17.3	55.2 ± 17.2	55.4 ± 21.5	< 0.001
Statin use	381,229 (7.6)	378,101 (7.6)	3153 (9.8)	< 0.001

Data are presented as mean ± standard deviation or median (interquartile range) for continuous variables and number (%) for categorical variables.

*TB* tuberculosis, *GFR* glomerular filtration rate, *HDL* high-density lipoprotein, *LDL* low-density lipoprotein.

### Total cholesterol level and TB risk

During an average of 8_._2 years of follow-up, 32,078 cases of TB occurred after the enforced 1-year lag time. As shown in Table 2, we found an inverse relationship between the total cholesterol level and the risk of TB. Compared with subjects in the highest quartile, those in the lowest quartile for total cholesterol had the highest risk of TB overall, even after adjusting for multiple clinical variables, including statin use (model 2) (adjusted hazard ratio [HR] = 1.35, 95% confidence interval = 1.31–1.39). In addition, there was an inverse relationship between the time to TB development and total cholesterol level (the time to TB development was 3.9 ± 2.4 years in Q1 [the lowest quartile] group, 4.0 ± 2.5 years in Q2 group, 4.1 ± 2.4 years in Q3 group, and 4.2 ± 2.4 years in Q5 [the highest quartile] group, respectively). The associations between the individual lipid levels and the risk of TB are summarized in Supplemental Table S1.

**Table 2:** TB risk by quartile of total cholesterol level.

	Quartile of TC level	Number at risk	Cases	Duration (PY)	IR (10,000 PY)	HR (95% CI)		
						Crude model	Model 1	Model 2
Overall	Q1	1,254,432	9546	10,225,250	9.3	1.35 (1.31–1.39)	1.34 (1.29–1.38)	1.35 (1.31–1.39)
	Q2	1,255,891	8045	10,301,441	7.8	1.13 (1.09–1.16)	1.15 (1.11–1.19)	1.15 (1.11–1.19)
	Q3	1,238,977	7362	10,173,668	7.2	1.04 (1.01–1.08)	1.06 (1.02–1.09)	1.05 (1.02–1.09)
	Q4	1,251,266	7125	10,269,940	6.9	Ref	Ref	Ref
**Age group**								
Age < 65 years	Q1	1,099,255	6185	9,071,739	6.8	1.40 (1.34–1.45)	1.33 (1.27–1.38)	1.34 (1.28–1.38)
	Q2	1,104,052	5223	9,133,888	5.7	1.17 (1.12–1.22)	1.15 (1.10–1.19)	1.15 (1.10–1.19)
	Q3	1,076,909	4697	8,915,017	5.3	1.08 (1.04,1.12)	1.07 (1.02–1.11)	1.07 (1.02–1.11)
	Q4	1,064,651	4304	8,809,225	4.9	Ref	Ref	Ref
Age ≥ 65 years	Q1	155,177	3361	1,153,511	29.1	1.51 (1.44–1.59)	1.24 (1.18–1.30)	1.27 (1.21–1.34)
	Q2	151,839	2822	1,167,553	24.2	1.25 (1.19–1.32)	1.1(1.044,1.16)	1.11 (1.05–1.17)
	Q3	162,068	2665	1,258,651	21.2	1.10 (1.04–1.16)	1.02 (0.97–1.07)	1.02 (0.96–1.07)
	Q4	186,615	2821	1,460,715	19.3	Ref	Ref	Ref
	*p* for interaction					0.048	< 0.001	< 0.001
**BMI group**								
BMI < 18.5 kg/m^2^	Q1	79,333	1116	634,825	17.6	0.92 (0.81–1.05)	1.32 (1.16,1.50)	1.32 (1.16–1.50)
	Q2	51,621	656	414,654	15.8	0.83 (0.72–0.95)	1.11 (0.96–1.27)	1.11 (0.97–1.27)
	Q3	33,575	448	268,167	16.7	0.87 (0.76–1.01)	1.03 (0.89–1.19)	1.02 (0.89–1.19)
	Q4	19,846	300	156,634	19.2	Ref	Ref	Ref
18.5 ≤ BMI < 23.0 kg/m^2^	Q1	598,823	52,57	4,880,828	10.8	1.09 (1.04–1.14)	1.35 (1.29–1.42)	1.36 (1.30–1.42)
	Q2	528,369	4160	4,327,498	9.6	0.97 (0.93–1.02)	1.16 (1.10–1.21)	1.16 (1.10–1.21)
	Q3	451,288	3550	3,695,960	9.6	0.97 (0.92–1.02)	1.07(1.02–1.124)	1.07 (1.02–1.12)
	Q4	370,748	2999	3,028,541	9.9	Ref	Ref	Ref
23.0 ≤ BMI < 25.0 kg/m^2^	Q1	273,821	1735	2,237,728	7.8	1.19 (1.11–1.27)	1.28 (1.20–1.37)	1.31 (1.23–1.41)
	Q2	304,809	1704	2,506,803	6.8	1.04 (0.98–1.11)	1.156 (1.08–1.24)	1.16 (1.08–1.24)
	Q3	320,026	1663	2,634,080	6.3	0.97 (0.91–1.03)	1.037 (0.97–1.11)	1.03 (0.97–1.11)
	Q4	333,575	1791	2,743,383	6.5	Ref	Ref	Ref
BMI ≥ 25 kg/m^2^	Q1	302,455	1438	2,471,869	5.8	1.24 (1.16–1.33)	1.17 (1.09–1.25)	1.18 (1.11–1.27)
	Q2	371,092	1525	3,052,485	5.0	1.07 (1.00–1.14)	1.09 (1.02–1.17)	1.10 (1.03–1.17)
	Q3	434,088	1701	3,575,460	4.8	1.02 (0.95–1.08)	1.05 (0.98–1.12)	1.05 (0.98–1.12)
	Q4	527,097	2035	4,341,382	4.7	Ref	Ref	Ref
	*p* for interaction					0.001	< 0.001	< 0.001
**Diabetes mellitus**								
Without diabetes mellitus	Q1	1,140,206	8035	9,336,142	8.6	1.36 (1.31–1.40)	1.40 (1.34–1.45)	1.41 (1.36–1.46)
	Q2	1,160,137	6888	9,542,782	7.2	1.14 (1.01–1.18)	1.17 (1.13–1.22)	1.17 (1.13–1.22)
	Q3	1,138,970	6231	9,376,755	6.6	1.05 (1.01–1.09)	1.06 (1.03–1.10)	1.06 (1.02–1.10)
	Q4	1,125,198	5876	9,261,763	6.3	Ref	Ref	Ref
With diabetes mellitus	Q1	114,226	1511	889,108	17.0	1.37 (1.27–1.48)	1.08 (1.00–1.17)	1.12 (1.04–1.21)
	Q2	95,754	1157	758,659	15.3	1.23 (1.14–1.33)	1.06 (0.97–1.14)	1.07 (0.98–1.15)
	Q3	100,007	1131	796,913	14.2	1.15 (1.06–1.24)	1.05 (0.97–1.13)	1.05 (0.97–1.14)
	Q4	126,068	1249	1,008,177	12.4	Ref	Ref	Ref
	*p* for interaction					0.002	< 0.001	< 0.001

Data are presented as numbers and ratios (95% CI), as appropriate.

Model 1 is adjusted for age, sex, smoking status, drinking habits, physical activity, BMI, diabetes mellitus, asthma or COPD, and GFR.

Model 2 is adjusted for age, sex, smoking status, drinking habits, physical activity, BMI, diabetes mellitus, asthma or COPD, GRF, and statin use.

*TC* total cholesterol, *PY* person-years, *IR* incidence rate, *HR* hazard ratio, *CI* confidence interval, *BMI* body mass index, *Q1* the lowest quartile, *Q4* the highest quartile *BMI* body mass index *COPD* chronic obstructive pulmonary disease, *GFR* glomerular filtration rate.

### Total cholesterol level and TB risk in subgroups

Table 2 shows the association between the total cholesterol level and TB incidence in subgroups. Across all subgroups, there was an inverse relationship between the total cholesterol level and TB risk. There were significant interactions between total cholesterol level and age, BMI, and DM. The association between total cholesterol level and age in the development of TB was more evident in the younger group (< 65 years) than in the older group (≥ 65 years) (*p* for interaction < 0.001 in model 2). The interaction between total cholesterol level and BMI in the development of TB showed a more substantial association in non-obese patients (BMI < 25 kg/m^2^) than in obese patients (BMI ≥ 25 kg/m^2^) (*p* for interaction < 0.001 in model 2). Also, the association between total cholesterol level and TB risk was more evident in patients without DM than in those with DM (*p* for interaction < 0.001 in model 2). We found no significant interactions between the total cholesterol level and the other covariates (sex, alcohol use, physical activity, smoking, and CKD) in the development of TB (data not shown).

### The effect of statin use on the association between total cholesterol level and TB risk

**Figure 1:**
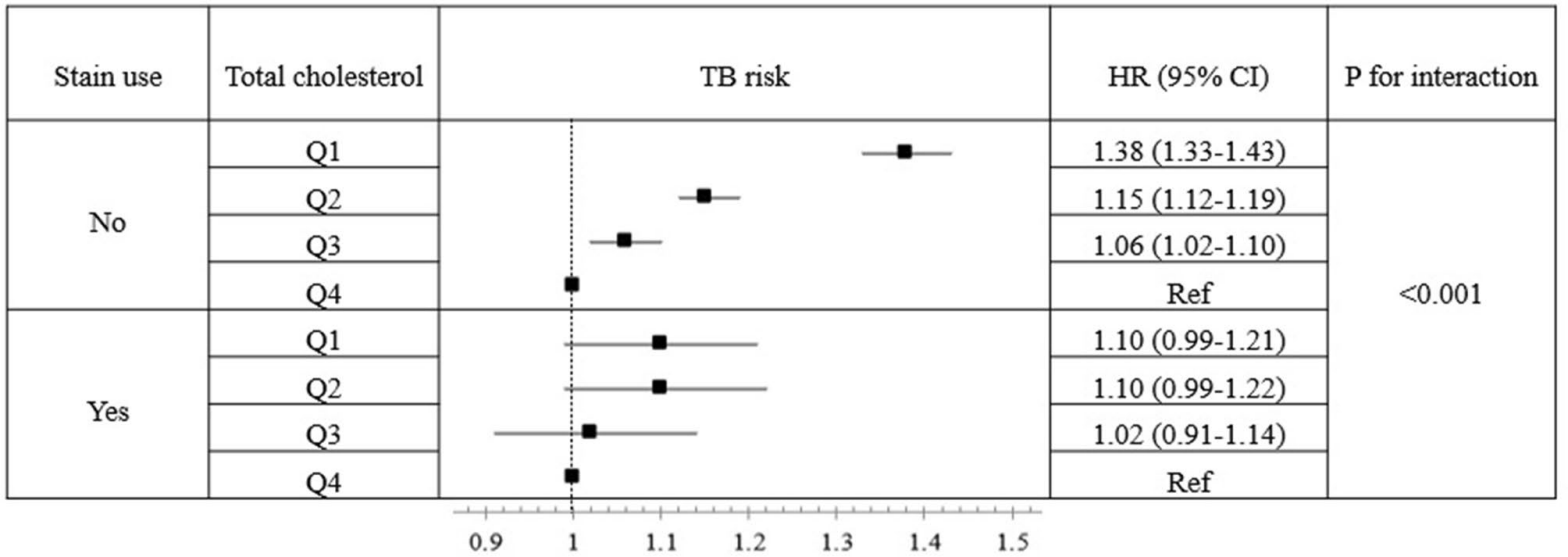
TB risk by quartile of total cholesterol level stratified by statin use. Age, sex, smoking status, drinking habits, physical activity, BMI, diabetes mellitus, asthma or COPD, and GFR were adjusted. *TB* tuberculosis, *HR* hazard ratio, *CI* confidence interval, *Q1* the lowest quartile, *Q4* the highest quartile, *BMI* body mass index, *COPD* chronic obstructive pulmonary disease, *GFR* glomerular filtration rate.

The relationship between the total cholesterol level and the development of TB according to statin use is presented in Fig. 1. In the crude model, there was an inverse relationship between the total cholesterol level and TB risk regardless of statin use. However, after adjustment for the covariables, the association between the total cholesterol level and TB risk differed significantly by statin use (*p* for interaction < 0.001). Among patients who did not receive a statin, there was an inverse association between total cholesterol level and TB risk, but that association lost its significance in patients who used a statin.

## Discussion

In this large population-based longitudinal study, we identified a clear relationship between a low total cholesterol level and high TB risk. The major strength of our study is that we used the largest-ever cohort with adequate lipid profile information. The inverse relationship between the total cholesterol level and TB risk was shown consistently in the subgroups, even after adjusting for potential confounding factors. In addition, we found that the effect of low total cholesterol on the development of TB was more robust in the following subgroups: younger age group, non-obese group, and non-DM group. When analyzed by statin use, whereas a significant inverse association existed between the total cholesterol level and TB risk in subjects not receiving statins, no significant association was observed between the total cholesterol level and TB risk in subjects receiving statins.

Some previous cross-sectional studies have suggested an inverse association between the total cholesterol level and TB^6–8^. According to those studies, patients with TB had significantly lower cholesterol levels than those without TB^6–8^, and anti-TB treatment improved their cholesterol levels^7,8^. However, those studies were limited by their cross-sectional designs and relatively small study populations. Furthermore, those studies did not consider confounding factors (e.g., low BMI^5^ and DM^10,11^) that can affect both lipid profiles and the development of TB. Therefore, the major strength of our study is that we clarified the inverse relationship between total cholesterol levels and TB risk in a longitudinal cohort study based on a large population, and we considered potential confounders.

The underlying mechanisms addressing the association between low cholesterol levels and TB risk are not well elucidated. Although the evaluation of those mechanisms is beyond our study scope, two explanations are plausible. First, a low cholesterol level could be a risk factor for TB. The innate and cell-mediated immune responses are key elements in the defense against TB^18^. The innate immune system recognizes *M. tuberculosis* through various pattern recognition receptors, which in turn activate phagocytosis, autophagy, and the inflammasome^18^. Activation of the cell-mediated immune system is known to promote the innate immune system (e.g., promotion of intraphagosomal killing of *M. tuberculosis* in macrophages)^18^. The role of cholesterol in the immune system is yet to be comprehensively revealed, but data indicate that it might have an important role in both innate and cell-mediated immunity. Cells depleted of cholesterol have dysfunctional pattern recognition–mediated immune activation^19,20^. In particular, the phagocytosis of *M. tuberculosis* by murine macrophages was shown to be significantly decreased when cholesterol was depleted^21^. Also, a recent study showed that a reduced cholesterol level was associated with T cell proliferation and function^22^. The second possibility is that a low cholesterol level might merely be a sign of TB during the latent period, instead of being a risk factor for TB. Our study design could not distinguish the former from the latter, and all epidemiological studies evaluating causal inferences harbor this limitation. Therefore, a well-designed study is needed to further evaluate the causal inference and underlying mechanisms of the association between cholesterol levels and TB risk.

In this study, the association between the total cholesterol level and TB risk was more robust in the younger age group, non-obese group, and non-DM group. Although we found a significant interaction between cholesterol levels and age, the difference in HR was small (HR = 1.34 in age < 65 years and HR = 1.27 in age ≥ 65 years) and might not be clinically relevant. It is well known that a high BMI is negatively associated with TB risk^5,23^. In this study, BMI had a significant interaction with the total cholesterol level in terms of TB risk. Although the underlying mechanisms are not well known, it seems that the protective effect of a high BMI against the development of TB reduced the effect that a low cholesterol level had on TB risk. In subjects with DM, the effect of the total cholesterol level on TB risk was not as apparent as in those without DM. The increased TB risk caused by DM^10^ itself seems to have a more profound effect than a low cholesterol level in subjects with DM. It is also likely that DM-associated conditions that increase TB risk, such as CKD^24^, affect the impact of a low cholesterol level on TB risk.

Interestingly, the apparent association between a low cholesterol level and TB risk was not found in patients taking a statin. As an explanation for that phenomenon, we suggest that the increased TB risk caused by statin-induced low cholesterol levels might have been offset by the immune system enhancement caused by statin use. For the disease progression of TB, the metabolism of macrophage cholesterol by *M. tuberculosis* is very important^17,25^. Because statins reduce macrophage cholesterol, as well as serum cholesterol^26^, the cholesterol metabolism by *M. tuberculosis* might be hindered by statins. Statins also have anti-inflammatory effects; recent studies showed that statins promote autophagy and phagosome activity in macrophages and enhance the secretion of proinflammatory cytokines by natural killer cells^27,28^.

The results of this study have several important clinical implications. First, low total cholesterol can be used as a predictor of TB in subjects already at high risk (e.g., subjects with a low BMI). Second, the results of our study should be interpreted cautiously. The inverse relationship between the total cholesterol level and TB risk in our study does not necessarily mean that a high-cholesterol diet can help to reduce TB risk in subjects with a high TB risk and low cholesterol level. Conflicting data have been published about the connection between a cholesterol-rich diet and TB. A population-based study in Singapore showed that a high cholesterol diet could increase TB risk^15^, and another study showed that a cholesterol-rich diet enhanced the sterilization of *M. tuberculosis* in the sputum of patients with pulmonary TB^29^. Accordingly, a well-designed study is needed to evaluate whether a high-cholesterol diet can prevent TB in high-risk subjects with low cholesterol. Third, our study results could be helpful to researchers investigating the mechanism of TB development by providing solid evidence about the inverse association between cholesterol levels and TB risk in humans.

Our study has several limitations. First, we used only one lipid profile per patient. Since not all participants were screened regularly as did in a prospective study, a study design including participants whose cholesterol levels were repeatedly measured was expected to cause several issues including selection bias and an increase in missing participants, which might decrease the statistical power of our study. However, as lipid profiles can vary over time, studies reflecting those variations are still needed. Second, we do not have detailed clinical information about TB, including the type and severity of the disease, because those details are not available in the Korean NHIS database. Third, our study was performed in a single country with an intermediate TB burden. Thus, our results might not be generalizable to the populations of countries with different TB burdens. However, our evaluation of a large number of subjects with lipid profile information is a strength of our study. Our study results could be beneficial to developing and developed countries with an intermediate TB burden.

In conclusion, we found an inverse association between total cholesterol levels and TB risk, and it was more robust in young subjects, those without DM, non-obese subjects, and those who did not use a statin. Future studies are needed to evaluate the underlying mechanisms by which cholesterol metabolism affects TB risk.

## Materials and methods

### Study population and design

We performed a population-based retrospective cohort study using information from the Korean National Health Insurance Service (NHIS) database^30^. In South Korea, a single-payer universal health system covers about 97% of all Korean citizens. Thus, the NHIS has claims data on every patient’s use of medical facilities, including International Classification of Diseases 10th Revision (ICD-10) codes, procedures, and prescriptions from outpatient clinics, emergency departments, and hospitalizations. Furthermore, the NHIS has socio-demographic data on all subscribers, including age, sex, and income level.

The NHIS database also contains data from annual or biennial health screening exams offered free of charge by the Ministry of Health and Welfare to all beneficiaries aged 40 years and older and all employees regardless of age. Approximately 72% of all eligible persons undergo this screening^31^, the screening data contain the answers to a self-reported questionnaire on health behavior (i.e., smoking status, alcohol consumption, physical activity, and past medical history), anthropometric measurements (e.g., BMI and blood pressure), and laboratory tests (e.g., hemoglobin, creatinine, and lipid profiles).

**Figure 2:**
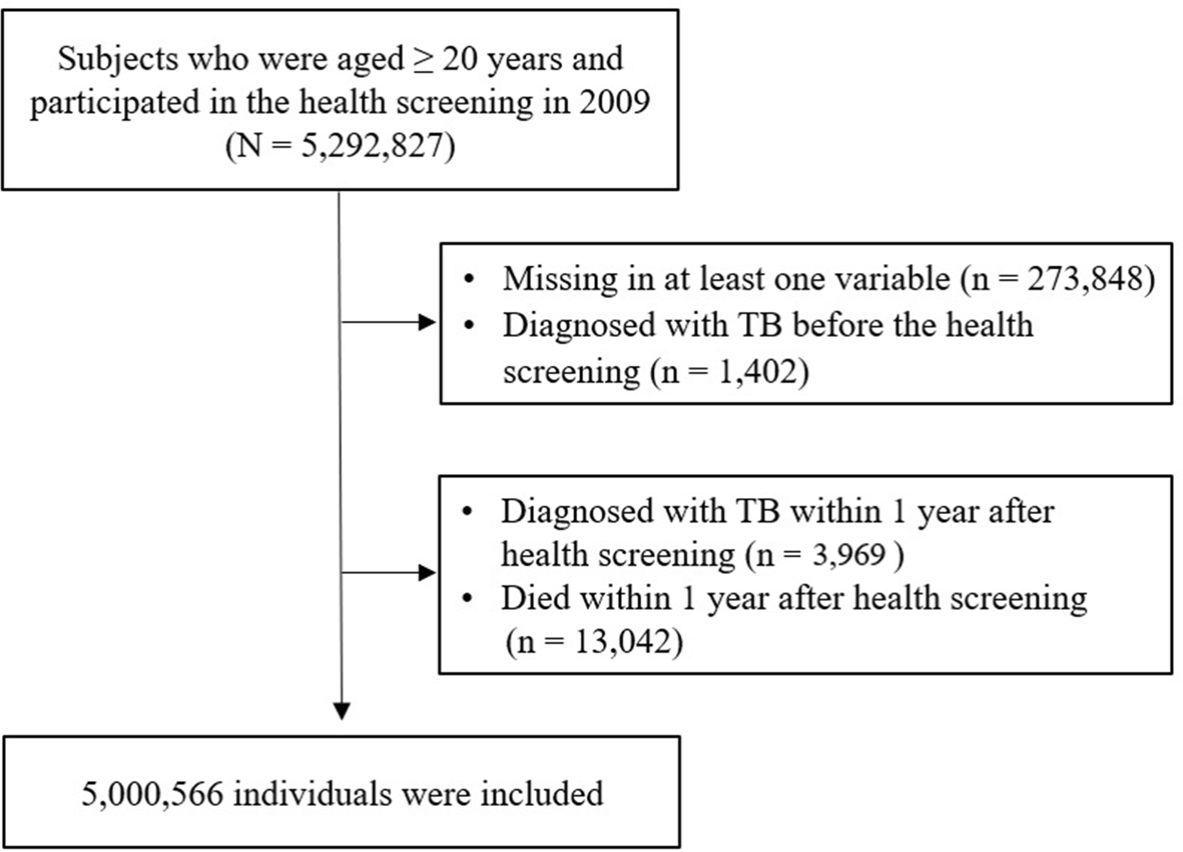
Flow chart.

This study initially included 5,292,827 subjects who were aged ≥ 20 years and received a health screening exam between January 1, 2009, and December 31, 2009. We excluded individuals who had missing data for any variable (n = 273,848), those who were diagnosed with TB before the health screening (n = 1402), those diagnosed with TB within 1 year after the health screening (n = 3969), and those who died within 1 year after the health screening (n = 13,042). A history of TB was defined by the following criteria: (1) presence of ICD-10 code for A15–A19, U880, or U881 at least twice in the 6 months between January 1, 2002, and December 31, 2008, or (2) findings of inactive TB, active TB, or suspected TB on chest radiography conducted as part of the health screening examination. In the end, we included 5,000,566 individuals in our analyses (Fig. 2). The cohort was followed from baseline to the date of incident TB or death or until the end of the study period (December 31, 2018), whichever came first.

### Definitions

#### Independent variable

For total cholesterol, LDL cholesterol, and triglycerides, the levels were classified and analysed by quartile (Q1—the lowest, Q2, Q3, and Q4—the highest). For high-density lipoprotein (HDL) cholesterol, the levels were classified and analysed by sex-specific quartile. The ranges of total cholesterol level were < 170 mg/dL in Q1, 171–192 mg/dL in Q2, 193–217 mg/dL in Q3, and ≥ 218 mg/dL in Q4; for LDL cholesterol, the ranges were < 91 mg/dL in Q1, 92–111 mg/dL in Q2, 112–134 mg/dL in Q3, and ≥ 135 in Q4; for triglycerides, the ranges were < 75 mg/dL in Q1, 75–108 mg/dL in Q2, 109–162 mg/dL in Q3, and ≥ 163 mg/dL in Q4; for HDL cholesterol in males, the ranges were < 44 mg/dL in Q1, 44–50 mg/dL in Q2, 51–59 mg/dL in Q3, and ≥ 60 mg/dL in Q4; for HDL cholesterol in females, the ranges were < 49 mg/dL in Q1, 49–56 mg/dL in Q2, 57–66 mg/dL in Q3, and ≥ 67 mg/dL in Q4.

#### Covariates

Based on the results from the self-administered questionnaire, the subjects were classified according to their physical activity as none, irregular, and regular. Regular physical activity was defined as performing > 30 min of moderate physical activity at least 5 times per week or > 20 min of strenuous physical activity at least 3 times per week. Smoking status was classified into never, former, and current smokers. The subjects’ rate of alcohol consumption was classified as none (0 g/day), moderate (< 30 g/day), and heavy (≥ 30 g/day).

BMI was calculated as the subject’s weight in kilograms divided by their height in meters squared, and the resulting BMI values were classified into 5 categories according to the Asia–Pacific criteria of the World Health Organization^32^: underweight (< 18.5 kg/m^2^), normal (18.5–23 kg/m^2^), overweight (23–25 kg/m^2^), and obese-to-severely obese (≥ 25 kg/m^2^). We used the Modification of Diet in Renal Disease equation to calculate the glomerular filtration rate values^33^.

Regarding comorbidities, COPD was defined as ICD-10 codes J42–J44 except for J43.0 (unilateral emphysema), and asthma was defined as ICD-10 codes J45–J46. Comorbid COPD and asthma were assessed during the enrollment period^34^.

The determination of income level was based on monthly insurance premiums because insurance contributions are determined by income level and not health risk in Korea**.** Low income was defined as the lowest quartile of monthly insurance premiums.

#### Study outcome

The main study outcome was incident active TB during the follow-up period. Since 2005, the Korean NHIS has provided a special co-payment reduction program for all patients diagnosed with active TB to decrease the burden of TB on the nation. Specific insurance codes (V000, V206, and V246) are required for patients with active TB after confirmation of their diagnosis, and TB patients then receive free TB-related medical care. Because the NHIS database contains complete information about insured medical services throughout the country, the claims database enabled us to review all patients with active TB in the nation using their unique insurance codes^35^.

### Statistical analysis

Continuous variables were compared using two-tailed Student’s t-testing, and categorical variables were compared using χ^2^ testing. The incidence rate of the primary outcome was calculated by dividing the number of incident cases by the total follow-up duration (10,000 person-years). Univariable and multivariable Cox regression analyses were performed to evaluate the HRs of TB incidence according to the quartiles of lipid profiles. Multivariable models included the following: (1) age, sex, smoking status, alcohol consumption, physical activity, BMI, DM, asthma or COPD, glomerular filtration rate (model 1); (2) the variables in model 1 and statin use (model 2). To examine possible effect modification by subject characteristics, we conducted stratified analyses by age (< 65 vs. ≥ 65 years), BMI, DM status, and statin use. All statistical analyses were performed using SAS version 9.4 (SAS Institute Inc., Cary, NC, USA), and a two-tailed *p* value < 0.05 was considered to indicate statistical significance.

### Ethics statement

This study was approved by the Institutional Review Board of Hanyang University Hospital (IRB No. 2020-11-019-001). The anonymity of the data is guaranteed, and no individually identifiable information is included; therefore, informed consent was waived in this study. The present study complied with the Declaration of Helsinki.

## Supplementary Information

Supplementary Information.

## Data Availability

The datasets generated and/or analysed during the current study are available from the corresponding author on reasonable request.

## Abbreviations

BMI: Body mass index
COPD: Chronic obstructive pulmonary disease
CKD: Chronic kidney disease
DM: Diabetes mellitus
HDL: High-density lipoprotein
ICD-10: International Classification of Diseases 10th Revision
HR: Hazard ratio
LDL: Low-density lipoprotein
NHIS: National Health Insurance Service
OECD: Organization for Economic Cooperation and Development
Q1: The lowest quartile
Q4: The highest quartile
TB: Tuberculosis
