# Supplementary material for: Relationship between total cholesterol level and tuberculosis risk in a nationwide longitudinal cohort
Source: Sci Rep. 2021 Aug 10;11:16254. doi: 10.1038/s41598-021-95704-1 (PMC8355278; doi:10.1038/s41598-021-95704-1)
Supplement: Supplementary file 1 — Supplementary Information. [file 41598_2021_95704_MOESM1_ESM.docx]

**Table S1.** TB risk by quartile of individual lipid profiles

| Quartiles of lipid profiles | Number at risk | Cases | Duration (PY) | IR  (10,000 PY) | HR (95% CI) | | |
| --- | --- | --- | --- | --- | --- | --- | --- |
|  |  |  |  |  | Crude model | Model 1 | Model 2 |
| LDL cholesterol |  |  |  |  |  |  |  |
| Q1 | 1,265,839 | 9,392 | 10,320,006.5 | 9.1 | 1.30 (1.265,1.345) | 1.32 (1.28-1.34) | 1.33 (1.29-1.38) |
| Q2 | 1,257,938 | 8,077 | 10,315,827.6 | 7.8 | 1.12 (1.087,1.16) | 1.17 (1.14-1.21) | 1.18 (1.14-1.21) |
| Q3 | 1,248,287 | 7,569 | 10,248,459.4 | 7.4 | 1.06 (1.03-1.09) | 1.09 (1.05-1.13) | 1.09 (1.05-1.12) |
| Q4 | 1,228,502 | 7,040 | 10,086,004.7 | 7.0 | Ref | Ref | Ref |
| Triglycerides |  |  |  |  |  |  |  |
| Q1 | 1,241,638 | 7,916 | 10,208,742.8 | 7.8 | 1.07 (1.04-1.10) | 1.11 (1.08-1.15) | 1.11 (1.08-1.15) |
| Q2 | 1,246,392 | 8,361 | 10,207,527.0 | 8.3 | 1.13 (1.09-1.16) | 1.04 (1.010-1.08) | 1.04 (1.01-1.08) |
| Q3 | 1,258,073 | 8,342 | 10,287,809.5 | 8.1 | 1.12 (1.08-1.15) | 1.02 (0.99-1.06) | 1.02 (0.99-1.06) |
| Q4 | 1,254,463 | 7,459 | 10,266,218.9 | 7.3 | Ref | Ref | Ref |
| HDL cholesterol |  |  |  |  |  |  |  |
| Q1 | 1,296,690 | 8,751 | 10,589,736.1 | 8.3 | 1.01 (0.98-1.04) | 1.05 (1.02-1.08) | 1.05 (1.02-1.08) |
| Q2 | 1,176,527 | 7,071 | 9,656,350.8 | 7.3 | 0.90 (0.87-0.92) | 0.97 (0.94-1.00) | 0.97 (0.94-1.00) |
| Q3 | 1,264,237 | 7,793 | 10,380,590.8 | 7.5 | 0.92 (0.89-0.95) | 0.99 (0.96-1.02) | 0.99 (0.96-1.02) |
| Q4 | 1,263,112 | 8,463 | 10,343,620.6 | 8.2 | Ref | Ref | Ref |

Data are presented as numbers and ratios (95% CI), as appropriate.

Model 1 is adjusted for age, sex, smoking status, drinking habits, physical activity, BMI, diabetes mellitus, asthma or COPD, and GFR.

Model 2 is adjusted for age, sex, smoking status, drinking habits, physical activity, BMI, diabetes mellitus, asthma or COPD, GFR, and statin use.

Abbreviations: LDL, low-density lipoprotein; HDL, high-density lipoprotein; IR, incidence rate; PY, person-years; HR, hazard ratio; CI, confidence interval; BMI, body mass index, COPD, chronic obstructive pulmonary disease; GFR, glomerular filtration rate.
